# First analysis of the secretome of the canine heartworm, *Dirofilaria immitis*

**DOI:** 10.1186/1756-3305-5-140

**Published:** 2012-07-10

**Authors:** James Geary, Mohamed Satti, Yovany Moreno, Nicole Madrill, Doug Whitten, Dalen Agnew, Timothy Geary, Charles Mackenzie

**Affiliations:** 1 Department of Pathobiology and Diagnostic Investigation, College of Veterinary Medicine, Michigan State University, East Lansing, MI 48824, USA; 2 Institute of Parasitology, McGill University, Ste-Anne-de-Bellevue, QC H9X 3V9, Canada; 3 Department of Plant Science, Michigan State University, East Lansing, MI 48824, USA; 4 School of Veterinary Medicine, St Mathew’s University, Orlando, Grand Cayman; 5 Harvard School of Public Health, Boston, MA 02115, USA; 6 Current address: Laboratory of Veterinary Pathology, Faculty of Veterinary Medicine, Universidade Norte do Paraná, Arapongas, PR, Brazil

**Keywords:** *Dirofilaria*, Heartworm, Canine, Feline, Nematode, Filarial, Secretome, Proteins

## Abstract

**Background:**

The characterization of proteins released from filariae is an important step in addressing many of the needs in the diagnosis and treatment of these clinically important parasites, as well as contributing to a clearer understanding of their biology. This report describes findings on the proteins released during * in vitro * cultivation of adult * Dirofilaria immitis *, the causative agent of canine and feline heartworm disease. Differences in protein secretion among nematodes * in vivo * may relate to the ecological niche of each parasite and the pathological changes that they induce.

**Methods:**

The proteins in the secretions of cultured adult worms were run on Tris-Glycine gels, bands separated and peptides from each band analysed by ultra mass spectrometry and compared with a FastA dataset of predicted tryptic peptides derived from a genome sequence of * D. immitis. *

**Results:**

This study identified 110 proteins. Of these proteins, 52 were unique to * D. immitis *. A total of 23 (44%) were recognized as proteins likely to be secreted. Although these proteins were unique, the motifs were conserved compared with proteins secreted by other nematodes.

**Conclusion:**

The present data indicate that *D. immitis* secretes proteins that are unique to this species, when compared with *Brugia malayi*. The two major functional groups of molecules represented were those representing cellular and of metabolic processes. Unique proteins might be important for maintaining an infection in the host environment, intimately involved in the pathogenesis of disease and may also provide new tools for the diagnosis of heartworm infection.

## Background

The filarial nematode *Dirofilaria immitis*, the aetiologic agent of heartworm infection in dogs and cats, is widely distributed in the United States, South America and parts of Europe and Asia [1]. The adult worms can be found mainly in the pulmonary arteries, and sometimes the right heart, atrium and vena cava in heavy infections; this differs from many other filariae that tend to favour lymphatic vessels. Infections with small numbers of adult *D. immitis* may be asymptomatic and have limited pathological effects; however, high adult worms loads usually cause exercise intolerance, a wet cough and lethargy in dogs [2]. Cats are inherently resistant to Dirofilarial infections and thus usually have much lower adult worm burdens than do dogs. However, as cats have a much smaller pulmonary arterial tree they are more susceptible to embolism. In addition, dirofilariasis in cats is often more difficult to diagnose due to lower loads and the differing clinical signs from those in dogs [2].

Although * D. immitis * has been controlled through several different strategies, the most successful has been the prophylactic administration of a range of drug combinations and administration schedules, most usually involving tablets or topical preparations containing a macrocyclic lactone (ML) anthelmintic to uninfected dogs and cats to protect them by killing infective L_3_ larvae and developing L_4_ larvae [3]; drugs in this class of agents are also microfilaricidal. MLs also affect adult worms, thus inducing long-term suppression in the production of microfilariae (mff) [4]. There are, however, concerns relative to the development of ML resistance [5,6]. A course of arsenical drugs, such as the currently preferred malarsomine, is adulticidal, although this regimen is not without risk to the animal due to the hepato- and nephron-toxicity of these compounds [7,8]; ‘slow-kill’ strategies for use of MLs in infected dogs have also been developed [9], and the potential for anti-*Wolbachia* treatment options to reduce transmission and pathological effects following adulticidal therapy is promising [10,11].

It has long been recognized that parasitic nematodes release factors, primarily proteins, which alter the immune responses of their hosts [12-14]. Recently, the use of sophisticated mass spectroscopy-based approaches, coupled to genome and transcriptome sequencing, has enabled the identification of proteins released by *Brugia malayi *[15-17] and *Heligmosomoides polygyrus polygyrus* (now considered to represent * H. bakeri *- [18]) into culture medium [19,20]. Secreted proteins have also been characterized from the canine hookworm, * Ancylostoma caninum *[21], the plant parasitic nematode * Meloidogyne incognita *[22] and from *Strongyloides stercoralis *[23]. Not all of these nematode datasets were analyzed against complete genomes (or transcriptomes), and, therefore, some of the compilations may be less completely assigned than others. Nonetheless, it can be concluded that a large number of proteins have been detected in the secretome from these parasites, with marked differences observed among them. The complexity of the nematode secretome compromises the ability to define the most biologically important proteins through a systematic analysis. One approach to provide some focus to this question is to define the secreted proteins that are conserved among parasites that share a niche (e.g., tissue *versus* gastrointestinal tract), and to consider those shared between phylogenetically related organisms (for instance those in Clade III vs. Clade V; [24]). Thus far, the data sets for nematodes are limited to parasites from different clades and different habitats. The present study describes the secretome of * D. immitis *, as distinct from that of * B. malayi, * which resides in a different niche in the mammalian host.

A more pragmatic reason to study the composition of parasite secretomes is to identify the most abundant proteins released into host fluids and tissues which could be candidates for the development of new diagnostic tests, and possibly new treatments. Current diagnostic procedures for nematodes typically rely on poorly characterized or proprietary antigens or antibodies, or the counting of eggs in faecal specimens: the identification of abundantly secreted proteins may allow the development of tests which can assess worm burdens, a goal not readily attainable using current diagnostic tools [25].

## Methods

### Parasite retrieval

Eighty mixed sex, adult * D. immitis * worms were collected from the pulmonary vessels and right heart chamber from mf test-positive dogs immediately after euthanasia, and the healthy worms placed in the culture fluid, as described below. These procedures were approved by the Animal Use Committee of St. Matthew’s University School of Veterinary Medicine (Grand Cayman, British West Indies). At the end of each 24 h period, immotile worms were removed from the culture system; thus, 56 worms were cultured on day 2 and 51 on day 3, the two days on which culture medium was collected for analysis.

### Parasite culture

Worms were cultured in large Petri dishes (1 worm/4 mL medium, 5 worms per dish) at 39°C in RPMI 1640 medium, supplemented with 200 mM L-glutamine, 20 mM HEPES, 200 IU/mL penicillin, 200 IU/mL streptomycin, 25 μg/mL amphotericin B (Gibco Invitrogen, Grand Island, NY), 1% w/v D-glucose and 1% w/v sodium bicarbonate, pH 7.2. Medium was collected and changed every 24 h. To limit potential contamination of the samples with host proteins, first-day medium was discarded. Medium from the subsequent 2 days was collected for molecular analysis. To determine the vitality of the worms, the change in colour (pH) of the medium was monitored to verify that all worms present were actively metabolizing. Petri dishes that exhibited a colour change were used for analysis, whereas those that remained unchanged or contained immotile worms were discarded. Based on this protocol, medium from 107 worm-days of cultures was collected. On the third day, the concentration was decreased to 1 worm/6 mL. Protease inhibitors (Complete EASYpack Roche, Indianapolis, IN) were added to batches of 50 mL of collected medium.

### Protein preparation

Immediately after the removal of adult worms, the medium samples were centrifuged at 1000 x *g* for 5 min to pellet mff released during the incubation. The supernatant was removed, passed through a 0.22 μm filter and frozen at −20°C for shipment to Michigan State University. There, the combined volume (775 mL) was concentrated to 40 mL using an Amicon Ultra 3000 MWCO (Millipore, Billerica, MA). Proteins were then precipitated using trichloroacetic acid (final concentration of 20%). Pelleted proteins were washed with cold (−20°C) acetone 3 times and allowed to air dry [15-17].

### Protein analysis

Protein pellets were dissolved in 100 μL sodium dodecyl sulfate – polyacrylamide gel electrophoresis (SDS-PAGE) sample buffer (50 mM Tris–HCl, 2% SDS, 10% glycerol,1% β-mercaptoethanol, 12.5 mM EDTA, 0.02% bromophenol blue, pH 6.8) and re-precipitated with chloroform:methanol (1:4). Pellets were re-solubilized in SDS-PAGE sample buffer and run on a BioRad Criterion precast 12.5% Tris-Glycine gel at 50 V for 15 min, followed by 120 V until the dye front reached the bottom of the gel (~ 90 min). The gel was fixed overnight in 40% methanol/20% acetic acid, followed by staining with colloidal Coomassie Blue. The entire gel lane was sectioned into 10 equal slices, and each slice was digested in-gel, essentially as described previously [26]. Briefly, gel bands were dehydrated using 100% acetonitrile and incubated with 10 mM dithiothreitol in 100 mM ammonium bicarbonate, pH ~ 8, at 56°C for 45 min, dehydrated again and incubated in the dark with 50 mM iodoacetamide in 100 mM ammonium bicarbonate for 20 min. Gel bands were then washed with ammonium bicarbonate and dehydrated again. Sequencing grade, modified trypsin was prepared to 0.01 μg/μL in 50 mM ammonium bicarbonate and ~50 μL were added to each gel band, so that the gel was submerged. Bands were incubated at 37°C overnight. Extracted peptides were re-suspended in 20 μL 2% acetonitrile/0.1% trifluoroacetic acid.

A 10 μL aliquot of each sample was automatically injected by a Waters nanoAcquity Sample Manager (http://www.waters.com) and loaded for 5 min on to a Waters Symmetry C18 peptide trap (5 μm, 180 um × 20 mm) at 4 μL/min in 2% acetonitrile /0.1% formic acid. Bound peptides were eluted using a Waters nanoAcquity UPLC (Buffer A = 99.9% water/0.1% formic acid, Buffer B = 99.9% acetonitrile/0.1% formic acid) onto a Michrom MAGIC C18AQ column (3 μm, 200 A, 100 U × 150 mm, http://www.michrom.com) and eluted over 90 min with a gradient of 5% B to 35% B in 78 min at a flow rate of 1 μL/min. Eluted peptides were sprayed into a ThermoFisher LTQ-FT Ultra mass spectrometer (http://www.thermo.com) using a Michrom ADVANCE nanospray source. Survey scans were taken in the FT (25000 resolution determined at m/z 400) and the top ten ions in each survey scan subjected to automatic low energy collision induced dissociation (CID) in the LTQ. The resultant MS/MS spectra were converted to peak lists using BioWorks Browser v3.3.1 (ThermoFisher) using the default parameters and the peptide masses were compared with a FastA dataset of predicted tryptic peptides derived from a genome sequence of * D. immitis * (P. Maser, personal communication;see http://nematodes.org/downloads/959nematodegenomes/blast/db/Dirofilaria_immitis_v1.3_20110901.fna) and the NCBI database, including the * Canis familiaris * genome, using the Mascot algorithm v2.3 (http://www.matrixscience.com). Search parameters were restricted to allow up to two missed tryptic sites, fixed modification of carbamidomethyl cysteine, variable modification of oxidation of methionine, peptide tolerance of +/− 10 ppm and MS/MS tolerance of 0.6 Da. The Mascot output was analyzed using Scaffold, v3.2, (http://www.proteomesoftware.com) to probabilistically validate protein identifications using the ProteinProphet algorithm [27]. Assignments validated above the Scaffold 95% confidence filter were considered true. The data have been submitted to Tranche.

### Data analysis

Blast2GO [28] was used to analyze the returned proteins as described elsewhere [15-17]. Briefly, an initial BLASTP search was performed against the non-redundant NCBI protein database. Subsequently, the annotation was completed using default parameters [29,30]. SecretomeP [31]: http://(http://www.cbs.dtu.dk/services/SecretomeP/) and SignalP [32]: http://(http://www.cbs.dtu.dk/services/SignalP/) were used to assess secretory motifs in the proteins.

## Results

### Amount of protein

**Figure 1:**
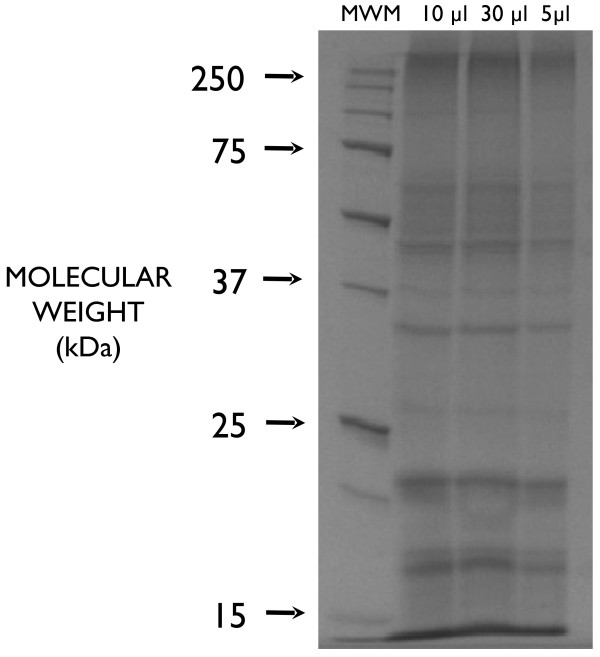
**SDS-PAGE gel of protein isolated from culture fluid derived from incubating mixed sex* Dirofilaria immitis *adult worms.** Lanes represent a Molecular weight marker (MWM) and 10, 30 and 5 μl amounts of the isolated protein.

Approximately 60 μg of protein was collected from the * D. immitis * cultures. An initial SDS-PAGE run revealed a complex pattern of proteins (Figure 1). The three lanes were combined for tryptic digestion and MS/MS analysis. A second gel consisted of a single lane that contained the same amount of protein analyzed in the first PAGE run.

### Protein analysis

The initial analysis revealed a total of 110 proteins. Results from the second run confirmed the first, with an additional 17 low abundance proteins appearing in this run. Following manual curation, redundant proteins (attributed to the same *D. immitis* locus) were removed, leaving a total of 110 proteins in this analysis of the heartworm secretome (Additional file 1: Table S1). Previous reports identified 193 proteins in the adult * B. malayi * secretome using similar methods [16].

Of these 110 proteins identified in the heartworm secretome, 52 were unique to *D. immitis*, not being described as being present in the *B. malayi* secretome [15-17]. Two of these proteins returned no BLAST hits, leaving 50 defined proteins unique in this context. The proteins found in common in the filariae were concentrated among the more abundant hits, but were distributed through the set, with the 15 most abundant proteins generally shared between the two filariae. Table 1 shows the 15 most abundant proteins detected in the heartworm secretome. The 15 most abundant proteins in the heartworm-unique sample are shown in Table 2. Of these unique defined proteins, 47 (90%) had Gene Ontology (GO) terms assigned in Blast2GO.

**Table 1:** **The 15 most abundant proteins detected in the* Dirofilaria immitis *secretome**

**SEQUENCE DESCRIPTION**	**ABUNDANCE RANK**
Phosphatidylethanolamine-binding protein	1
Ladder protein	2
Ml domain containing protein	3
Transthyretin-like protein 5	4
Glycosyl hydrolases family 31 protein	5
Transthyretin-like protein 5	6
LL20 15kda ladder antigen	7
Transthyretin-like protein partial	8
Plasma glutamate carboxypeptidase-like	9
NADH-dependent fumarate reductase	10
Cysteine protease inhibitor	11
Abhydrolase domain containing isoform cra_a	12
Transthyretin-like protein partial	13
Immunogenic protein 3	14
Exocyst complex component 2	15

**Table 2:** **The 15 most abundant unique proteins in the* Dirofilaria immitis *secretome**

**SEQUENCE DESCRIPTION**	**ABUNDANCE RANK**
Abhydrolase domain containing isoform cra_a	12
Glutathione s-transferase 1	18
Elegans protein partially confirmed by transcript evidence	22
cre-pqn-85 protein	22
Nipped-b-like protein	22
Pdz domain containing protein	26
Jheh1	27
Alpha-actinin	30
Epoxide hydrolase 1	32
Protein dek isoform 1	35
Kh domain containing protein	37
Protein szt2	38
Hypothetical protein LOAG_04081 [Loa loa]	39
Elongation factor tu homologue precursor	40
Pan domain containing protein	44

**Figure 2:**
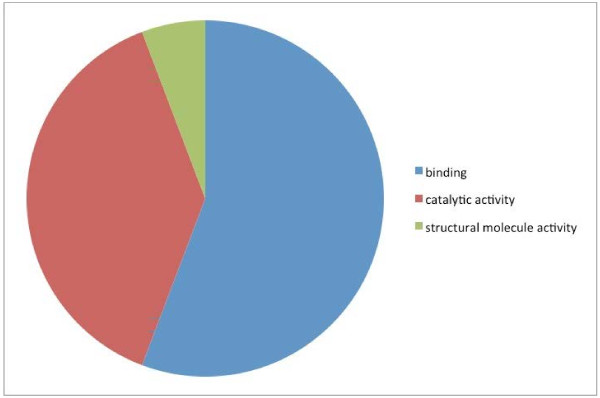
*** Dirofilaria immitis *protein analysis: Distribution of the most abundant (level 2) molecular functions using GO terms.**

**Figure 3:**
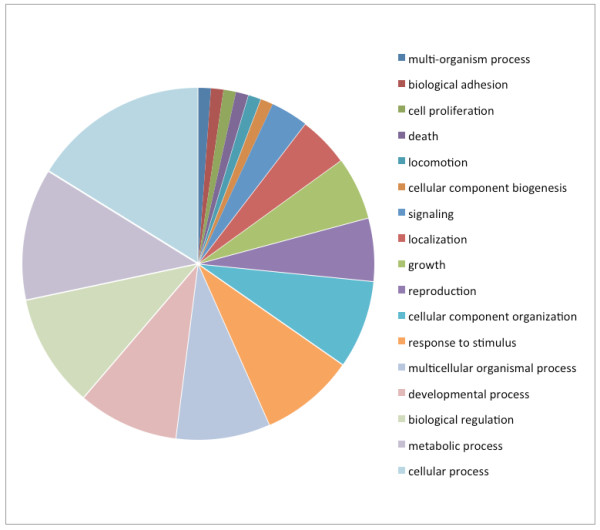
*** Dirofilaria immitis *protein analysis: Distribution of the most abundant (level 2) biological process using GO terms.**

**Figure 4:**
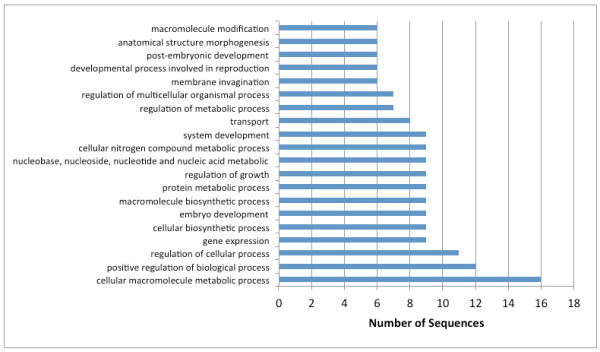
*** Dirofilaria immitis *protein profile.** Distribution of the top 20 most abundant (level 4) biological processes (GO terms).

**Figure 5:**
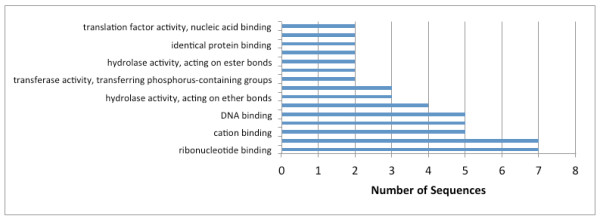
*** Dirofilaria immitis *protein profile.** Distribution of the most abundant (level 4) molecular functions (GO terms).

Catalytic activity (GO:0003824) and binding (GO:0005488) were the two major molecular function categories, those using GO terms (Figure 2) and those according to biological process (Figure 3). This distribution was highly conserved with that reported for * B. malayi *[16]. The distribution of level 4 biological process GO terms was fairly flat (Figure 4), with no marked bias for particular functions. The category of ‘cellular macromolecule metabolic process’ was the most frequent term in this category, while ‘ribonucleotide binding’ was the most frequent term in the set of returned level 4 molecular function GO terms (Figure 5). For both, the distribution of term frequency was generally similar to those reported for *B. malayi *[16].

Analysis for secretion signals by Secretome P and Signal P showed 10 (19%) of the *D. immitis* unique proteins include a canonical signal sequence for secretion. An additional 13 (25%) include peptide sequences associated with non-classical secretion pathways, for a total of 23 (44%) that can be recognized as proteins likely to be secreted in some manner. This figure is somewhat less than the corresponding percentages previously reported for *B. malayi *[16].

Several mammalian proteins, clearly not of nematode origin, were also detected in the protein samples (data not shown). These proteins included keratin, titin and serum albumin, among others, but did not interfere with the characterization of the heartworm secretome. No bacterial proteins were present.

## Discussion

The initial analysis revealed a secretome consisting of 110 proteins, identified through the analysis of ~30 μg protein collected during cultivation of adult heartworms. MS/MS analysis of the same amount of protein was repeated independently on the second gel; these results confirmed the first, with 17 additional low-abundance proteins being displayed in this run; manual curation revealed that 17 of the identified proteins were duplicates. The high agreement between independent replicates of the MS/MS analysis suggests that a reliable and reasonably complete accounting of the proteins present in this sample was obtained, in consideration of the amount of protein available and the intrinsic sensitivity of the methods. The 110 proteins identified in these two experiments (Additional file 1: Table S1) were combined for further analysis. Using similar methods, 193 proteins were reported in the adult * B. malayi * secretome from ~ 100 μg of protein, suggesting that the procedures generated similar efficiencies of protein recovery and identification [16].

Of the 110 proteins identified in the heartworm secretome, 52 were not present in the published secretome of * B. malayi *[15-17]. The degree of relatedness of the secretome composition of these two species was higher than that of either with the secretomes of the other nematode species for which comprehensive datasets are available (not shown) [18-22]; since these data were generated using different methods and produced quite different numbers of identified proteins from multiple developmental stages, a detailed species-species comparison is unwarranted at this time. As an example, however, the comparative data reveal that the secretomes of the filarial species are much more closely related (53% identical) than either is to * H. polygyrus, * a gastrointestinal nematode in a different clade (V * versus * III; <20% identical). It is possible to discern a set of 17 secretome proteins which are common to species that parasitize mammals, including * D. immitis * (Table 3), and so constitute a minimal consensus secretome of species from distinct clades [23] which inhabit different niches as adults. All of these proteins, except cystatin, macrophage migration inhibition factor, triosephosphate isomerase and phosphatidylethanolamine-binding protein, have also been detected in the * M. incognita* secretome [21]. The functions embodied in this list can, in general, be associated with roles in modifying host responses or in protein-release pathways. The inclusion of additional nematode species in secretome analyses will enable this list to be refined, but the available data suggest that secretome composition may be highly adapted to the site of residence of these parasites. It is interesting, in this regard, that we did not find in the * D. immitis * secretome some classes of proteins which have been reported in the secretomes of both other nematodes, including * B. malayi *, such as a variety of proteases and globins. Whether their absence from the current secretome is due to the lower amount of heartworm protein available for this analysis or to a fundamental difference in the menu of secreted proteins among these species requires additional research.

**Table 3:** **Proteins found in the* Dirofilaria immitis * secretome with functions that are known to be usually conserved in nematodes**

**SEQUENCE DESCRIPTION**	**ABUNDANCE RANK**
Phosphatidylethanolamine-binding protein	1
Transthyretin family proteins	4
LL20 15 kDa ladder antigen	7
Cysteine protease inhibitor/cystatin	11
Immunogenic protein 3	14
Glutathione-S-transferase	18
Lectins (galectin/galactoside-binding protein)	20
Actin	23
Enolase	42
Triosephosphate isomerase	46
Macrophage migration inhibition factor	50
Heat shock protein 70	52
Fatty acid-bunding protein	54
Protein disulfide isomerase	77
Cyclophilin	61
Fumarase	90
Aldolase	100

List compiled from published secretomes [15-18,28-30].

The proteins identified in common in the filariae were distributed throughout the data set. However, the shared proteins were much more likely to be among the most, as opposed to the least abundant molecules, indicating a high correlation between proteins secreted in abundance by the two filariae. Two of the * D. immitis *-unique proteins returned no BLAST hits, leaving 50 defined proteins unique, in this context, in the * D. immitis * secretome. Of these proteins, 45 (87%) could be assigned GO terms in Blast2GO. Catalytic activity (GO:0003824) and binding (GO:0005488) were the two major molecular function categories (Figures 2 &3), while cellular (GO:0009987) and metabolic (GO:0008152) processes were the two major biological process categories (Figures 2 &3) for the heartworm-unique proteins. This distribution was, in general, quite conserved with that reported for proteins in the * B. malayi * secretome using similar methods [16]. Consideration of the functions of the heartworm-unique secretome did not identify any molecules with special relevance to the niche inhibited by this species, compared with that of *B. malayi*.

About 40% of the heartworm-unique proteins contained amino acid sequences that are associated with classical or non-classical secretion pathways. This figure is somewhat lower than the corresponding figure (~ 65%) reported for *B. malayi* using similar methods [16]. An explanation for this discrepancy is not readily apparent; additional data on the identification of proteins released in the host (as opposed to in culture) could resolve the biological relevance of their detection in these experiments. It should also be noted that many secreted proteins are now recognized as being released in exosomes, which represent a significant pathway in eukaryotic and prokaryotic organisms [33,34]. Indeed, exosomes-mediated secretion events have been detected in the * C. elegans * excretory canal [35]. Evidence is not available on the anatomical localization of exosomes in parasitic nematode secretory systems, but many proteins detected in nematode secretomes, including actin, elongation factors, aldolase, enolase, HSP70 and cyclophilin, are common components of mammalian exosomes (http://www.exocarta.org/exosome_markers). A recent report identified 27 * Onchocerca ochengi * proteins recovered from nodules [36], including many homologs of secretome proteins in other filariae. The majority of the * O. ochengi * proteins lacked secretion signals and are associated with exosomes in other organisms; these data support the relevance of the antigens detected * in vitro * and suggest that the role of exosomes, as a source of secreted proteins, warrants further investigation.

The impact of * D. immitis * infections on companion animal health and veterinary practice in endemic areas cannot be overstated. In endemic areas, the prevalence of infection can be as high as 20% in areas in which prophylaxis treatment is irregular [1]. The development and introduction of the highly efficacious and relatively inexpensive ML-based regimens for prophylaxis have produced one of the most successful mass drug administration programs in history. However, there are emerging concerns of resistance to the MLs most commonly used for heartworm prevention [6,7]. Data obtained from this experiment may assist in addressing this situation in several ways. First, current methods of testing for prophylactic activity against * D. immitis * are exceptionally time-constrained, as they monitor the onset of microfilaremia in treated dogs, which occurs ~ 8 months after infection [1]. A biomarker based on abundantly secreted proteins might allow detection of worms that survive the prophylactic regimen shortly after infection, and the proteins reported here are candidates for the development of such a test. Similarly, a number of current diagnostic tests based on antigen detection have been advanced for the diagnosis of *D. immitis* infection, but all of them have some problems with sensitivity and none is associated with a reported parasite protein [37]. A legitimate goal is the development of a test that can accurately predict adult worm burdens [1,38], which can be an important factor in deciding on a course of treatment for infected animals. A test based on the most abundantly secreted parasite proteins may be better able to fulfil that role. Antigen-based diagnostic tests for human filarial infections have similar limitations, including the lack of well-described antigens in some tests, which have not been selected based on abundance in serum, concerns about sensitivity and an uncertain correlation with adult worm burden [39-43].

From a therapeutic standpoint, efforts to limit survival or development of heartworms with immunological interventions, such as vaccination, could be enhanced if proteins essential for the success of an infection were targeted as vaccine antigens. Previous work in this area seems to have been typically focused on parasite proteins that generated significant immune responses in dogs [44], which is not necessarily a predictor of value as a protective antigen. Instead, down-stream experimental work on the functional role of secreted proteins could identify candidates for which a strong antibody response would prevent establishment of an infection. A menu of secreted proteins, provided here, is essential for that work to proceed. This same line of reasoning suggests that the proteins identified may yield novel therapeutic targets. At least some of these secreted proteins may be critical for the survival of the parasite within the host. Any interference with their function * via * the administration of a therapeutic antibody may have a detrimental effect on the parasite’s ability to remain viable, offering a possible alternative to the current arsenical-based strategy to cure established infections [1]. As the composition of the secretome varies according to life-cycle stage and sex in *B. malayi *[16,17], it will be important to determine the contribution to the current * D. immitis * secretome from male, female and mff before advancing into new research in this area.

The data obtained from this experiment yielded some information on putative functions of these proteins, which may help to illuminate the difference between the niches exploited by the various worms whose secretomes are compiled. As * D. immitis * resides in the bloodstream of the host, it is reasonable to expect some level of difference between both the gut-dwelling * H. polygyrus * and the lymph-residing * B. malayi *, which can be seen in these data. Of the 110 identified proteins, 52 (47%) were unique to * D. immitis * compared to the nematode secretomes compiled previously.

It is known that a number of proteins are commonly conserved across nematodes species and are from this current study are also found in * D. immitis * (Table 3). A few of the proteins characterized as unique share a common family with proteins secreted by * B. malayi *. For instance, galectin was highly abundant in the *B. malayi* secretome, but the closest homolog in the *D. immitis * secretome was a galactoside-binding lectin family protein, which presumably has a similar or related functional role. However, a BLASTp analysis revealed that the *D. immitis* genome encodes a predicted protein that is almost identical to the * B. malayi * galectin (not shown), but which was not secreted proteins. Similarly, the glutathione s-transferase 1 found in the heartworm secretome was related to a homolog identified in the *B. malayi* secretome [16-18], but the closest homolog of the * B. malayi * protein in the * D. immitis * genome (data not shown) was not found in the current study. The implications of these findings are not clear; the functional conservation of these protein families in the secretomes of the two filarial species does not account for the discrepancy in secretion of the most closely related proteins between the two species.

A hypothesis driving investigations into the composition of the * D. immitis* secretome is that at least some of them should be adapted for the task of living in blood. Protease inhibitors and proteins that detoxify oxygen radicals are likely important for any parasite in a host; candidates specifically pertinent for life the bloodstream are not readily apparent. The family of transthyretin-like proteins is highly represented in the heartworm secretome. This family is represented by a large number of genes in *C. elegans *[45], the functions of which are largely unknown. However, transport functions have been associated with this family [45], and it would be advantageous to study their biological function in this regard in nematodes in general and tissue-dwelling species in particular.

The anatomy of secretory apparatuses in adult *D. immitis* is unknown. In general, the adult filariid secretory system is either glandular or tubular. In each kind of system, a duct links the secretory cells and opens to the exterior through a secretory pore that may be muscularly controlled [46]. In addition to a discrete secretory compartment analogous to that found in mff [6], proteins may be discharged into the medium from uterine fluid during the release of mff by females, from the release of cuticle-associated materials or from defecation of incompletely digested parasite or host proteins. Several canine proteins were detected in these samples; whether they arose from incomplete washing of the worms or from excretion *via* the faecal route cannot be concluded. The contribution of proteins secreted *versus* those excreted or discharged into the medium (e.g., intestinal waste and/or uterine fluid) could be resolved by further experiments.

## Conclusions

This is the first report of the secretory proteome of * D. immitis *, which lives in the circulatory system rather than the lymphatic vessels (* B. malayi *). Adult * D. immitis * were collected from dogs immediately after euthanasia and cultured for 3 days in RPMI 1640 media. This media was processed and yielded 110 proteins, 52 of which have not been reported in the secretomes of any other nematodes studied to date. Although these proteins were unique, their functional categories and motifs are generally similar to those of proteins released by other nematode species.

## Abbreviations

ML, Macrocyclic lactone; HARD, Heartworm Associated Respiratory Distress; mff, Microfilariae; GO, Gene ontology; HEPES, (4-(2-hydroxyethyl)-1-piperazineethanesulfonic acid); NCBI, National Center for Biotechnology Information; SDS-PAGE, Sodium dodecyl sulfate-polyacrylamide gel electrophoresis; LC-MS/MS, Liquid chromatography tandem mass spectroscopy.

## Competing interests

The authors declare that they have no competing interests.

## Authors’ contributions

JG carried out preparation of the parasite material, the proteome analysis and the writing of the manuscript. MS, DA, NM, YM and SAH were all involved in the collection and preparation of the parasite material. TG was involved in the conception of the project, and CDM the project conception, collection of parasite material and the writing of the manuscript. All authors read and approved the manuscript.

## Supplementary Material

Additional file 1 **Table S1** The secretome of *Dirofilaria immitis.* Click here for file
